# Both Isocarbohydrate and Hypercarbohydrate Fruit Preloads Curbed Postprandial Glycemic Excursion in Healthy Subjects

**DOI:** 10.3390/nu13072470

**Published:** 2021-07-19

**Authors:** Xuejiao Lu, Jiacan Lu, Zhihong Fan, Anshu Liu, Wenqi Zhao, Yixue Wu, Ruixin Zhu

**Affiliations:** 1 College of Food Science and Nutritional Engineering, China Agricultural University, Beijing 100083, China; feirlu@163.com (X.L.); jiacanlu0613@163.com (J.L.); liuanshu@cau.edu.cn (A.L.); zhaowenqi@cau.edu.cn (W.Z.); xiaoc0105@126.com (Y.W.); zhuruixin07@126.com (R.Z.); 2 Key Laboratory of Precision Nutrition and Food Quality, Department of Nutrition and Health, China Agricultural University, Beijing 100083, China

**Keywords:** glycemic response, preload, fruit, apple, satiety

## Abstract

This study aimed to investigate the impact of fruit preloads on the acute postprandial glycemic response (PGR) and satiety response of a rice meal in healthy female subjects based on iso-carbohydrate (IC) and hyper-carbohydrate (HC) contents, respectively. The IC test meals including (1) rice preload (R + 35R), (2) orange preload (O + 35R), (3) apple preload (A + 35R) and (4) pear preload (P + 35R), contained 50.0 g available carbohydrates (AC) where the preload contributed 15.0 g and rice provided 35.0 g. The HC meals included (1) orange preload (O + 50R), (2) apple preload (A+50R) and (3) pear preload (P + 50R), each containing 65.0 g AC, where the fruits contributed 15.0 g and rice provided 50.0 g. Drinking water 30 min before the rice meal was taken as reference (W + 50R). All the preload treatments, irrespective of IC or HC meals, resulted in remarkable reduction (*p* < 0.001) in terms of incremental peak glucose (IPG) and the maximum amplitude of glycemic excursion in 180 min (MAGE_0–180_), also a significant decrease (*p* < 0.05) in the area of PGR contributed by per gram of AC (AAC), compared with the W + 50R. Apple elicited the lowest PGR among all test meals, as the A + 35R halved the IPG and slashed the incremental area under the curve in 180 min (iAUC_0–180_) by 45.7%, while the A + 50R reduced the IPG by 29.7%, compared with the W + 50R. All the preload meals and the reference meal showed comparable self-reported satiety in spite of the difference in AC. In conclusion, pre-meal consumption of three fruits effectively curbed post-meal glycemia even in the case of a 30% extra carbohydrate load.

## 1. Introduction

White rice, a high glycemic index (GI) food [1,2], is the most popular staple food in Asian cultures. Daily intake of high GI staple foods such as white rice may lead to large postprandial glycemic fluctuation [3] even in healthy people [4]. Results from several meta-analyses suggested that a positive association between the consumption of white rice and the prevalence of type 2 diabetes [5,6].

Increasing evidence indicated that some macronutrients preload could stabilize postprandial glycemic response (PGR) and improve HbA1c in long-term treatments [7,8,9,10,11]. Whey protein preload could blunt the postprandial glucose excursion partly by enhancing the insulin response and slowing gastric emptying [8,12,13]. However, a few studies suggested that pre-meal consumed carbohydrates exerted a hypoglycemic effect as well, such as small doses of fructose [14], glucose [15] and mixed glucose–fructose solution [16].

The World Health Organization (WHO) called for a limitation of free sugar [17], as the consumption of refined sugar would result in a reduction of nutrient density, especially the excessive consumption of fructose from the sugar-sweetened beverages concerning a series of adverse health outcomes [18,19,20]. However, the sugar in a reasonable amount of fruit is not a concern, as fruit consumption increases the intake of phytochemicals, potassium and dietary fiber, which are beneficial to the prevention of cardiovascular diseases [21,22,23].

Moreover, increasing epidemiological studies and meta-analyses have reported that daily consumption of fruit has relevance to a lower incidence of type 2 diabetes [24,25,26,27,28]. It is reported that partial substitution of white rice with certain fruits or dried fruits improved PGRs [16,29,30] and HbA1c levels [31].

In a previous study, on the equi-carbohydrate basis, a 30% substitution of rice meal with apple preload halved the PGR of a rice meal in an acute test [16]. However, given the scarcity of fruit preload studies, there are more questions to be answered. (1) The simple replacement of rice by apple inevitably led to less protein intake, which would account for the magnitude of PGR and satiety response. What will be the glycemic effect of partially replacing rice meals with fruit preload based on controlled protein intake? (2) Most people are used to enjoying fruit after or between meals without reducing the carbohydrate quantity of the meals, which may result in more calories and a higher glycemic peak [32]. Will the effect of apple preload remain when it is added to a rice meal as an extra carbohydrate load rather than an equi-carbohydrate substitution? (3) Can the hypoglycemic power of fruit preload be generalized to more fruits other than apple and kiwifruit? (4) Compared with high GI starchy foods in daily meals, foods characterized with low energy density and high fiber content are expected for improved satiety [33,34,35]. If the fruits are given as an extra carbohydrate load, will they elicit greater satiety in line with the energy compensation logic?

In this study, apples, oranges and pears were selected to evaluate the PGR and satiety response of fresh fruits preload to a rice meal with three research hypotheses. (1) Any fruit preload would have some hypoglycemic effect to a rice meal, regardless of the type of the fruit, while the extent of the effect might be different. (2) The fruit preload treatments would curb the glycemic excursion after a rice meal even when the fruit preload was given as an extra carbohydrate load. (3) The satiety response would not be significantly affected by the extra carbohydrate preload.

## 2. Materials and Methods

### 2.1. Materials

Red Fuji apple (*Malus pumila* Mill.) was produced in Yantai, Shandong Province, China. Hosui pear (*Pyrus pyrifolia* Nakai.cv.Hosui) was produced in Liaocheng, Shandong Province, China. South Africa’s orange (*Citrus sinensis* (Linn.) Osbeck) was produced in South Africa. White rice (*Oryza sativa spp*. japonica) was cultivated and milled in Jilin, Jilin Province, China. Hen egg and sesame oil were produced in Beijing, China, bought in the local supermarket.

### 2.2. Subjects

Generally healthy participants aged 18–25 years old college students were recruited through bulletin boards, online advertisements and moments. The subjects were screened according to the following inclusion criteria: (1) free from food allergies and intolerance; (2) with a body mass index (BMI) between 18.5 and 23.9 kg/m^2^; (3) weight stable (±2 kg) within the last six months; (4) not on diet to gain or to lose weight in the past three months; (5) having three meals regularly; (6) no abnormal blood biochemical test record; (7) no ongoing medical condition or treatment; (8) normal glucose tolerance; (9) a regular menstrual cycle (if women); (10) not pregnant or lactating; (11) no tendency of eating disorders; (12) no diagnosed digestive system diseases, or self-reported frequent gastrointestinal upset; (13) no dependency on caffeine-containing or alcoholic beverages; (14) no dependency on drugs; (15) non-smoker. The self-reported biochemical data were obtained from the physical examination record of the university.

The sample size calculation was done with the PASS 13 *Power Analysis and Sample Size* software (NCSS, Kaysville, UT, USA). Assuming that the standard deviation (SD) is lower than 55.15 mmol·min/L, a sample size of *n* = 11 provided 80% power to detect a change of 167.8 mmol·min/L in iAUC (*p* < 0.05), based on our previous study [16].

Participants interested in the study were screened on the basis of the inclusion criteria and then involved in duplicated oral glucose tolerance tests (OGTT), blood pressure measurement and body composition analysis in the laboratory. Ethics approval was obtained from the China Agricultural University Ethics Committee (ethics number CAUHR-2019006) and all eligible individuals provided the informed consent forms. The trial was carried out at the College of Food Science and Nutritional Engineering, China Agricultural University, being in accordance with the Declaration of Helsinki.

### 2.3. Study Design

This study was designed as an acute randomized, crossover trial where participants were randomly assigned to 8 test meals on 8 separate occasions according to the sequence performed by a computer random number generator, with at least one week apart to ensure adequate washout. Subjects were instructed to not consume any fruits or fruit products, and refrain from coffee, tea or alcohol, as well as excessive consumption, intensive exercise and later bedtime on the day prior to each study session. A visit on another day was required if subjects were within 3 days prior to and after the start of menstruation, or did not keep to the above-mentioned requirements.

### 2.4. Test Meals

Eligible subjects underwent 8 test meals consisting of 3 groups: reference, iso-carbohydrate test meals (IC) and hyper-carbohydrate test meals (HC). In the non-preload reference meal, drinking water was given 30 min before the consumption of white rice containing 50.0 g available carbohydrates (AC) (W + 50R). There was a 30 min-interval between the preload food and rice meal in both IC and HC groups. IC were designed to contain 50.0 g AC in total, where the fruit or the rice preload contributed 15.0 g and the rice provided 35.0 g: (1) rice preload (R + 35R); (2) orange preload (O + 35R); (3) apple preload (A + 35R); (4) pear preload (P + 35R). HC were given containing a total of 65.0 g AC where the fruit preload contributed 15.0 g and the rice provided 50.0 g: (1) orange preload (O + 50R); (2) apple preload (A + 50R); (3) pear preload (P + 50R). The composition and nutrient content of the test meals are shown in Table 1.

**Figure 1:**
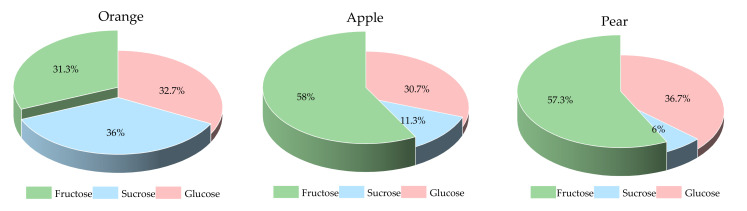
The sugar fractions of the three fruits.

The sugar fractions of the three fruits are shown in Figure 1. The pear and apple had a higher proportion of fructose than the orange did. In a 15.0 g AC portion of preload fruits, the contents of fructose, sucrose and glucose were 4.7, 5.4 and 4.9 g for orange, 8.7, 1.7 and 4.6 g for apple, and 8.6, 0.9 and 5.5 g for pear, respectively.

### 2.5. PGR Assessment

On each test day, the subjects came to the laboratory at 8:15 a.m. after a 12 h overnight fast and had two fasting plasma glucose samples at −10 min and 0 min after a short rest. Then the preload food or water was provided to the subjects at 0 min and the food was ingested within 5 min. The white rice was served 30 min after the start of the preload and was ingested within 10 min. Then 200 mL of water at room temperature was supplied at 120 min and was consumed before the end of the test. An additional 8 blood samples at 15, 30, 45, 60, 90, 120, 150 and 180 min were obtained by finger-prick, measured by glucometer (LifeScan Inc., Milpitas, CA, USA) using the glucose oxidase method, with the first drop of blood discarded.

### 2.6. Satiety Assessment

The subjective satiety during each test session was assessed by visual analog scale (VAS) [36], which is a 100-mm rating scale with the statement “extremely hungry” on one end and “extremely full” on the other end. The subjects ranked their level of satiety at 0, 15, 30, 45, 60, 90, 120, 150, 180 min, also at the end time of preload food and rice meal consumption.

### 2.7. Primary Outcome and Statistical Analysis

The fasting glucose for each subject was taken as the mean value of the glucose concentrations at −10 min and 0 min. The glycemic data analysis was based on the glucose change value relative to the fasting glucose. The incremental areas under the curve (iAUC) of PGR were calculated using the trapezoidal rule, ignoring the area beneath the fasting glucose level. The incremental peak glucose concentrations (IPG) and the maximum amplitudes of glucose excursion in 180 min (MAGE_0–180_) were defined. In order to adjust the amount of available carbohydrates of the test meals, the area of PGR contributed by per gram of available carbohydrate (AAC) was defined to predict the change of glycemic parameters, where AAC = iAUC_0–180_ (mmol·min/L)/the available carbohydrates of test meals (g).

The satiety data analysis was based on the VAS change value relative to the baseline. The incremental areas under the curve (iAUC) of postprandial satiety responses and the incremental peak of satiety (IPS) were calculated. In order to adjust the energy intake of the test meals, the peak satiety density (PSD) was defined to predict the change of satiety, where PSD = IPS (mm)/the energy of test meal (MJ).

The statistical analysis was performed by an investigator blinded to the study group, using the SPSS version 21.0 (SPSS Inc. Chicago, IL, USA). Two-factor repeated-measures ANOVA assessed the effects of treatment × time on PGR and satiety. One-way analysis of variance ANOVA and Duncan’s multiple range test were performed to compare the effects of test meals on the above-mentioned characteristic values of PGR and satiety response. The variables are presented as the mean ± standard deviation (SD) or the mean value with standard error (SE), with *p* < 0.05 considered statistically significant.

## 3. Results

### 3.1. Subject Characteristics

**Figure 2:**
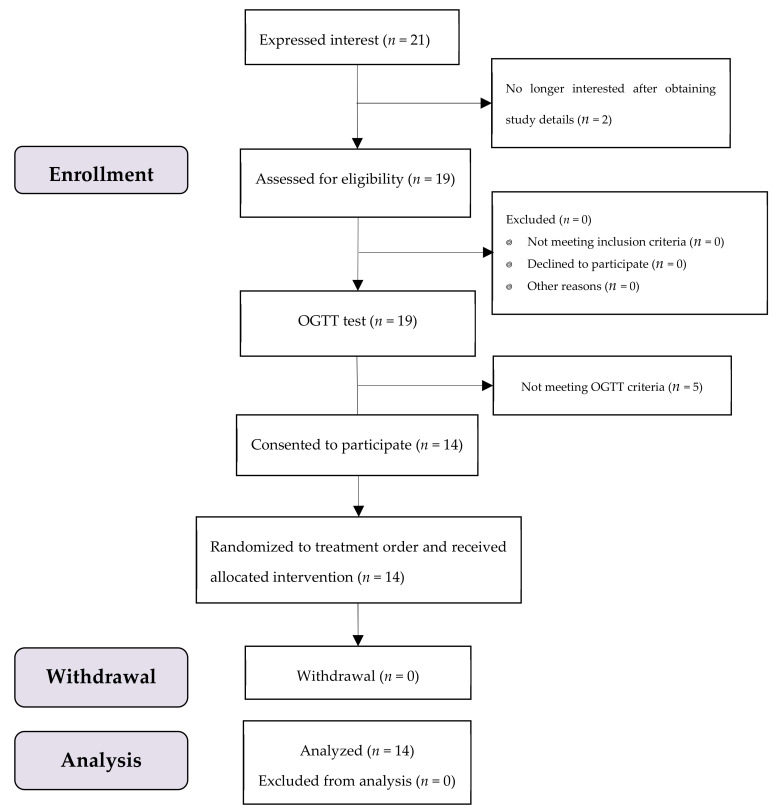
The study subjects flow diagram.

A total of 19 participants were enrolled, where 14 subjects met the OGTT criteria with no one dropped out throughout the test period (Figure 2). All of them were female university students aged 22.0 ± 1.3 (mean ± SD) years, with a mean (SD) BMI of 19.2 (1.2) kg/m^2^. All data of 14 subjects were included in the analysis.

### 3.2. PGR

**Figure 3:**
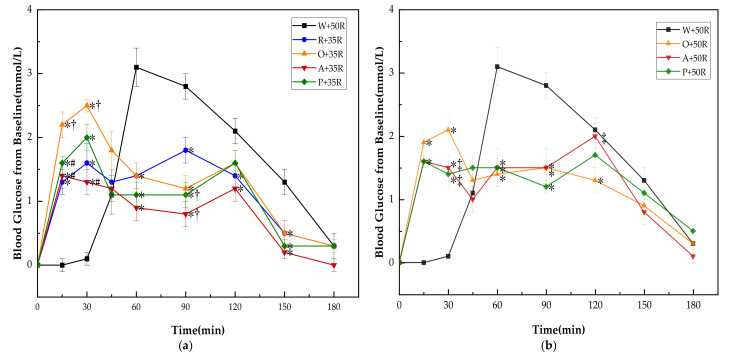
(**a**) PGRs for reference and IC test meals; (**b**) PGRs for reference and HC test meals. G, glucose; R, rice; O, oranges; A, apples; P, pears; W, water. Data are presented as the mean values with their standard errors, *n* = 14. ^*^ Test meals different from W + 50R, ^†^ Test meals different from R + 35R, ^#^ Test meals different from O + 35R, ^‡^ Test meals different from O + 50R (*p* < 0.05).

There was no significant difference in baseline (i.e., 0 min) blood glucose between all the test meals. The PGRs for all test meals are shown in Figure 3. The PGR pattern of preload meals was characterized by double small peaks instead of one sharp peak in the case of the W + 50R. One of the peaks appeared immediately following the preload, while another peak showed up long after the rice meal. Though the preloads led to higher glucose levels at 15 and 30 min, all of them dramatically reduced the postprandial glucose at 60 and 90 min (*p* < 0.001) in both IC and HC groups, and at 150 min in the IC group (*p* < 0.01). In the IC group (Figure 3a), the R + 35R and A + 35R cut down the blood glucose level at 120 min compared with W + 50R (*p* < 0.05). The P + 35R and A + 35R further lowered the glucose level at 90 min compared with R + 35R (*p* < 0.05). In the HC group (Figure 3b), the O + 50R also cut down the glucose level at 120 min compared with W + 50R (*p* < 0.05). Among the preload meals, the orange preload showed the highest incremental values at 15 and 30 min in both IC and HC groups.

### 3.3. PGR Characteristics

As shown in Table 2, A + 35R showed the lowest iAUC_0–60_ among all preload test meals. All the preload test meals elicited smaller iAUC_60–120_ than W + 50R did (*p* < 0.001), but only the A + 35R had a significant lower iAUC_60–120_ than R + 35R (*p* < 0.01). The preload meals decreased the iAUC_120–180_ significantly compared with W + 50R (*p* < 0.05) in the IC group, but not in the HC group. In terms of iAUC_0–180_, A + 35R was the lowest and achieved a 45.7% and 29.1% decrease compared with W + 50R (*p* < 0.001) and R + 35R (*p* = 0.055), respectively.

Compared with the W + 50R, all the preload meals except for O + 35R achieved a significant decrease in AAC (*p* < 0.05), which represents the area contributed by per gram of available carbohydrate. The AAC of A + 35R was the smallest and achieved a 46.4% and 30.2% decrease compared with that of the W + 50R (*p* < 0.001) and R + 35R (*p* < 0.05), respectively.

**Figure 4:**
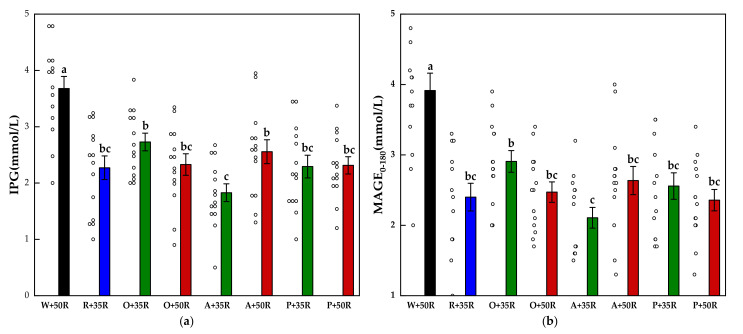
(**a**) IPG, (**b**) MAGE_0–180_ for test meals. The hollow circles indicate the data of each subject, the columns indicate the mean value, the error bar indicates the SE value. Significant differences (*p* < 0.05) are represented by different letters.

The IPG and MAGE_0–180_ for all test foods are shown in Figure 4. The IPG and the MAGE_0–180_ of the preload test meals were significantly lower than that of the W + 50R (*p* < 0.001). Compared with the W + 50R, the A + 35R had a 51.3% and 46.1% reduction in the IPG and MAGE_0–180_, respectively. The A + 35R also attained a significant decrease (*p* < 0.01) compared with O + 35R in terms of both the IPG and the MAGE_0–180_.

### 3.4. Subjective Satiety

**Figure 5:**
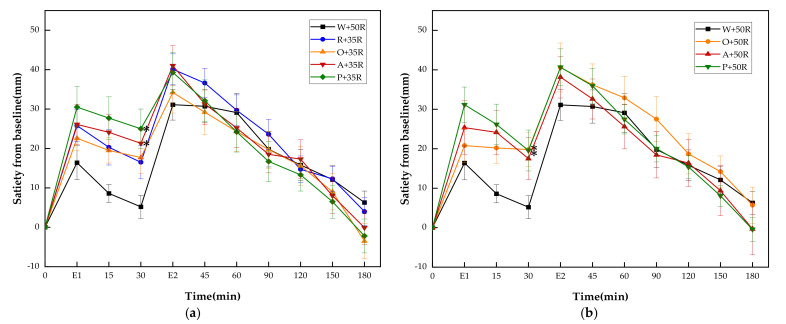
(**a**) Satiety responses for reference and IC test meals; (**b**) satiety responses for reference and HC test meals. E1, the end point of preload consumption; E2, the end point of rice consumption. G, glucose; R, rice; O, oranges; A, apples; P, pears; W, water. Data are given as mean ± SE, *n* = 14. ^*^ Test meals different from W + 50R.

As shown in Figure 5, the A + 35R, P + 35R, O + 50R and P + 50R showed significantly higher incremental satiety values than W + 50R only at 30 min. There were no differences at any other time point between W + 50R and the test meals of the HC group.

There were no significant differences between all test meals in any of the satiety characteristics except for PSD. The P + 50R showed a significant reduction of PSD compared with W + 50R and A + 35R (*p* < 0.05).

## 4. Discussion

In the present study, the apple and pear were chosen because both of them are among low GI temperate fruits associated with reduced risk of diabetes [32]. Apples are especially well-known for being a rich source of flavonoids, phenolic acids, anthocyanidins and pectin [37], which might add points to glycemic homeostasis in the long run [38,39]. Orange is a number of citrus fruits, which are rich sources of vitamin C, carotenoids and phytochemicals contributing to the prevention of diabetes and diabetic complications [40,41]. The tropical fruits such as durian, mango and banana were not included in the trial as the intake of tropical fruits was reported to be positively associated with the risk of diabetes in the Asian population [32].

In this study, all the preload test meals led to a sharp decrease in PGR, regardless of the preload manner, i.e., IC or HC. Even when loaded with additional 15 g AC, all fruit preloads significantly resulted in lower IPG and MAGE_0-180_ than the W + 50R control meal did, suggesting that fruits ingested 30 min prior to a high carbohydrate meal could be explored as a simple and practical hypoglycemic strategy.

It is important to verify the effect of the extra fruit preload because most people would not balance their carbohydrate intake of a meal after enjoying pre-meal fruits. The iAUC_0–180_s of HC test meals (containing 65 g AC) were not significantly lower than that of the control group (W + 50R, containing 50 g AC), but the area of PGR contributed by per gram of AC (AAC) of the preload meals achieved a significant reduction. Nevertheless, the hypoglycemic effects varied in degree despite the same carbohydrate, protein and fat contents in the IC test meals.

The IC tests reproduced the result in the previous study [16] that the apple preload halved the iAUC_0–180_, IPG and MAGE_0–180_. However, when preloading orange, the corresponding reduction was merely 16.5%, 27.0% and 25.6% respectively, compared with that of the W + 50R control. The result of the pear preload was in between that of the apple and orange.

The disparity of PGR of fruits preload in the IC group may be partly attributed to the fructose content. Although a large amount of fructose was implicated for playing a role in the development of diabetes [42], several meta-analyses indicated that energy-matched substitution of fructose (especially from fruit) for refined starches could improve HbA1c without any negative metabolic effects [43,44,45]. Previous literature suggested the ‘catalytic’ fructose doses (≤36 g/d) might be beneficial [46], and a range of 7–10 g fructose was reported to be able to promote glucose metabolism and improve PGR in some studies [14,47,48,49]. The fructose content of orange accounts for only 31% of its soluble sugar, while the apple and pear account for more than 55%. The fructose provided by the apple and pear preload was 8.7 g and 8.6 g, respectively, while the orange was only 4.5 g, falling out of the reported effective range of 7–10 g.

Growing evidence indicates that dietary fruit consumption is conducive to the prevention of type 2 diabetes [26,27,28,50]. However, the key component and biological mechanism responsible for the beneficial effects of fruits on PGR is yet to be elucidated. A study claimed that the sugar component extracted from kiwifruit failed to reduce the PGR of a co-ingested high GI meal as the kiwifruit did [51]. However, when given as a preload, the apple sugar solution was 39% as effective as an apple in terms of lowering the glycemic iAUC of rice meals [16].

It is possible that several factors contributed to the striking hypoglycemic effect of fruit preloads: (1) the preload effect, which could also be elicited by sugar or starch; (2) the action of an appropriate amount of fructose, which improved the hepatic glycogen metabolism [52,53]; (3) the effect of the phytochemicals in the fruits, which might retard the process of digestion and improve the insulin sensitivity [54,55]; (4) the fiber and texture of the fruits, which might delay the gastric emptying [56].

A number of sugar-containing preloads, including fructose [14], glucose [15], a mixture of fructose and glucose [16], fruits [16,29], dried apple [57], and vegetable drinks [58,59], were reported to be able to alleviate postprandial glycemic excursion to some degree. In the present study, rice preload of 15 g AC (R + 35R) also achieved a considerable drop of the IPG and MAGE_0–180_ compared with the W + 50R control meal. This result confirmed the preload effect of starchy food reported in previous studies [29,60].

It is interesting that, after the preload meals, the PGR curves manifested a small-double-peak curve instead of the one-sharp-peak curve in W + 50R control. This unique pattern is in line with the result of our previous study [16] and an earlier report [15]. The second peak would appear at 30–45 min after the ingestion of the high GI meals in glucose [15], starch preload [60] and vegetable drinks [58,59], but in the present study, the second peaks were delayed to 60–90 min.

A few preload studies investigated the insulin and incretin pattern of carbohydrate preloads. After 20 g glucose preload, the insulin level did elevate right after the ingestion but the postprandial peak value had no significant difference with that of the control meal [15]. The rice porridge and the kiwifruit preload meal elicited no significant increase in terms of the iAUCs of postprandial insulin, glucagon and ghrelin, compared to the control [29]. Hence, though there were no metabolic markers other than blood glucose in the present study, it is not reasonable to attribute the carbohydrate preload effect on glucose metabolism to a substantial increase of insulin. A possible explanation of the preload action, such as the “early onset of insulin” or the “improved insulin sensitivity” hypotheses, need to be tested in a future study.

The pre-meal apple treatment highlighted a combined action of the fructose, the phytochemicals, and the texture factors, in addition to the preload effect. Compared with the R + 35R, the iAUC_60–120_ and AAC of A + 35R decreased significantly. The IPG and MAGE_0–180_ of A + 35R were the lowest, 21.7% and 12.5% lower than that of R + 35R. In addition, the iAUC_0–180_ of A + 35R was 29.1% lower than that of R + 35R. It is noticed that the apple had higher fructose content than orange and was chewier than pear. A previous study reported that chewy texture and slower eating rate may increase the release of GLP−1 and peptide YY (PYY), which play a role in enhancing the insulin action [61,62].

With respect to the timing of fruit intake on the satiety of a meal, the results were inconsistent in limited studies. A previous study reported that consuming fruit before a meal reduced the subsequent energy intake by 166 kcal [63], while another study found that co-ingestion of apple and rice elicited a greater satiety response compared to the corresponding preload meal [16]. The present study found that the iAUC_0–180_ of all other test meals, either the HC or IC groups, had no significant difference with that of the W + 50R. There was no significant difference in the satiety measured by VAS between the HC (65 g AC) and the W + 50R control (50 g AC), suggesting an inadequate energy compensation in the case of extra sugar preload. It is noticed that the PSD of A + 35R was significantly higher than that of the P + 50R, in spite of the smaller bulk volume (134.8 g vs. 240.4 g), less rice and the similar fructose contents. The chewy texture and high pectin content in apples might play a role in its higher satiety response compared with pear. Given that daily fruit intake is beneficial to the prevention of chronic disease and some cancers [21], taking fruit as a preload of carbohydrate-dense meals is only a matter of timing instead of a risk of increasing energy intake.

To the best of our knowledge, this is the first study to investigate the effect of different types of fresh fruit preload on postprandial glycemia and satiety based on IC and HC (50 g and 65 g AC) modes. In this study, the macronutrient contents in iso-carbohydrate test meals and the meal size were carefully controlled to minimize possible confounding factors. However, this study is an acute test in healthy subjects. The effect of fruit preload in the prediabetic and the diabetic is still to be explored in a long-term intervention study. The exact mechanism of the hypoglycemic effect of carbohydrate preload needs to be elucidated by investigating the pattern of insulin and other gut hormones, the rate of gastric emptying and digestion, as well as the regulation of metabolic pathways.

## 5. Conclusions

In conclusion, the present study demonstrated that fruit preloads containing 15 g AC, either in iso-carbohydrate or hyper-carbohydrate manner, dramatically improved the PGR and reduced the glycemic excursion. Compared with the orange and pear, the apple preload performed the best in curbing the IPG and the MAGE_0–180_. The result of the study encourages further exploration of the hypoglycemic effect of fruit preload for people in need of PGR management.

## Figures and Tables

**Table 1:** Composition and nutrient content of test meals (per serving) ^1^.

Sample	Polished Rice (g)	Fruit (g)	Egg White ^2^ (g)	Sesame Oil ^3^ (g)	AC ^4^ (g)	Protein (g)	Fat (g)	Dietary Fiber (g)	Meal Size ^5^ (g)	Energy (kcal)
W + 50R	143.1	-	-	-	50.0	7.0	0.6	1.00	383.5	845.6
R + 35R	143.1	-	2.7	2.5	50.0	9.7	2.1	1.00	336.3	961.5
O + 35R	93.3	191.6	-	-	50.0	9.7	2.1	32.46	336.3	981.8
A + 35R	93.3	134.8	3.5	0.5	50.0	9.7	2.1	12.48	336.3	931.2
P + 35R	93.3	240.4	2.6	-	50.0	9.7	2.1	20.88	336.3	1147.1
O + 50R	143.1	191.6	-	-	65.0	11.8	2.3	32.46	383.5	1232.6
A + 50R	143.1	134.8	-	-	65.0	8.3	1.7	12.48	383.5	1121.9
P + 50R	143.1	240.4	-	-	65.0	9.2	2.3	20.88	383.5	1352.8

^1^ Nutrient content data were acquired through manufacturers and determination experiments. ^2^ Egg white was used to balance the protein contents in the IC group. ^3^ Sesame oil was used to adjust the fat contents in the IC group. ^4^ AC, available carbohydrate. ^5^ The water for weight balance was included. R, rice; O, oranges; A, apples; P, pears; W, water.

**Table 2:** PGR characteristics of test meals in 180 min (*n* = 14).

Sample	iAUC_0–60_		iAUC_60–120_		iAUC_120–180_		iAUC_0–180_		AAC	
	Mean	SE	Mean	SE	Mean	SE	Mean	SE	Mean	SE
W + 50R	44.8 ^a^	4.9	159.8 ^a^	7.3	75.3 ^a^	9.0	279.8 ^a^	8.9	5.6 ^a^	0.2
R + 35R	73.6b ^c^	9.3	96.0 ^b^	10.0	44.6 ^bc^	8.9	214.2 ^abc^	23.5	4.3 ^b^	0.5
O + 35R	106.9 ^d^	8.0	81.3 ^bc^	9.8	45.3 ^bc^	7.4	233.5 ^ab^	22.2	4.7 ^ab^	0.4
A + 35R	65.0 ^ab^	6.2	57.1 ^c^	8.6	29.7 ^c^	5.5	151.8 ^c^	16.3	3.0 ^c^	0.3
P + 35R	79.6 ^bc^	7.7	73.1 ^bc^	11.3	41.9 ^bc^	7.0	194.5 ^bc^	22.6	3.9 ^bc^	0.5
O + 50R	90.4 ^cd^	9.1	86.0 ^bc^	9.0	51.6 ^abc^	7.6	228.0 ^ab^	23.3	3.5 ^bc^	0.4
A + 50R	72.3 ^bc^	8.5	97.3 ^b^	11.8	57.0 ^ab^	8.3	226.5 ^ab^	24.8	3.5 ^bc^	0.4
P + 50R	78.2 ^bc^	6.4	84.2 ^bc^	8.2	66.2 ^ab^	9.0	228.6 ^ab^	20.5	3.5 ^bc^	0.3

G, glucose; R, rice; O, oranges; A, apples; P, pears; W, water. ^a,b,c,d^ Different superscript letters denote that mean values within a column are significantly different (*p* < 0.05).

## Data Availability

The data presented in this study are available on request from the corresponding author.
